# Roles of Interferons in Pregnant Women with Dengue Infection: Protective or Dangerous Factors

**DOI:** 10.1155/2017/1671607

**Published:** 2017-09-07

**Authors:** Hao Zhang, Zhiyi He, Wenting Zeng, Hong-Juan Peng

**Affiliations:** ^1^Department of Infectious Diseases, The Key Discipline of Guangdong Province, The First Affiliated Hospital of Guangzhou Medical University, Guangzhou Medical University, No. 151 Yanjiang Road, Guangdong Province 510120, China; ^2^Department of Obstetrics and Gynecology, The First Affiliated Hospital of Guangzhou Medical University, Guangzhou Medical University, No. 151 Yanjiang Road, Guangdong Province 510120, China; ^3^Guangdong Provincial Key Laboratory of Tropical Disease Research and Key Laboratory of Prevention and Control for Emerging Infectious Diseases of Guangdong Higher Institutes, School of Public Health and Tropical Medicine, Southern Medical University, No. 1023 South Shatai Road, Guangzhou, Guangdong Province 510515, China

## Abstract

Dengue infection is a serious public health problem in tropical and subtropical areas. With the recent outbreaks of Zika disease and its reported correlation with microcephaly, the large number of pregnancies with dengue infection has become a serious concern. This review describes the epidemiological characteristics of pregnancy with dengue and the initial immune response to dengue infection, especially in IFNs production in this group of patients. Dengue is much more prevalent in pregnant women compared with other populations. The severity of dengue is correlated with the level of IFNs, while the serum IFN level must be sufficiently high to maintain the pregnancy and to inhibit virus replication.

## 1. Background

Dengue is a febrile disease that is transmitted by* Aedes* mosquitoes. It is estimated that approximately 3900 million people in 128 countries are at risk of infection by dengue viruses [1]. This virus infects approximately 390 million people per year, 96 million of whom present disease [2]. The causative agent of dengue is dengue virus (DENV), which belongs to the family Flaviviridae. The clinical phenotype of dengue varies from a self-limiting febrile illness to severe, occasionally life-threatening disease. Typically, symptomatic disease follows three phases: a febrile phase lasting 3 to 7 days; a critical phase characterized by defervescence, during which complications appear in a small proportion of patients; and a spontaneous recovery phase. Complications primarily affect the vascular system and include an unusual plasma leakage syndrome that may result in dengue hemorrhagic fever (DHF) and dengue shock syndrome (DSS), which are classified as severe dengue according to the WHO guidelines for dengue diagnosis, treatment, and prevention [3, 4].

In 2015-2016, Zika virus caused large outbreaks in Pacific areas and in Central and South America [5]. Increasing data have shown that Zika virus infection is associated with adverse pregnancy outcomes such as fetal death, premature birth, and microcephalus [6]. Thus, DENV infection which belongs to the same family of Flaviviridae as Zika virus has become a serious concern in pregnant women.

## 2. Dengue in Pregnant Woman

With an estimated yearly case number of 390 million from more than 100 countries and regions in Asia, Oceania, America, and Africa, dengue fever (DF) is now one of the most important vector-borne diseases worldwide [2]. As reported in recent studies, a high rate of dengue infection has been observed in pregnant women. An investigation of 358 pregnant women in Malaysia, a dengue endemic country, showed that a seropositivity for dengue infection of approximately 35.8% by ELISA [7]. Additionally, in another dengue endemic country, Thailand, the dengue seroprevalence can reach 90.3% [8]. It must be noted, however, that these data were collected during the Chikungunya disease outbreak [8], raising the possibility of false positivity. In the African country, the Democratic Republic of Sao Tome and Principe, 39.74% of pregnant women were found to be positive for DENV antibodies [9]. These findings indicate that pregnant women have high a risk of dengue virus infection.

However, the prognosis for the pregnant women infected with dengue remains unknown. There might be two explanations. One explanation is that dengue infection is aggravated in pregnant women. Adam et al. reported that, among 30 pregnant women infected by dengue virus, approximately 38.3% of them developed dengue hemorrhagic fever or dengue shock syndrome, and the mortality could reach 21.7% [10]. Machado et al. also reported that 46.5% of pregnant women infected by dengue virus developed severe dengue, as compared with 22.5% of nonpregnant women [11]. These reports indicated that pregnant women infected with dengue virus had a greater tendency to experience illness progression to severe dengue and higher mortality, as compared with infected nonpregnant women. The other explanation is that adverse pregnancy outcomes emerge after infection. A recent meta-analysis indicated that common adverse outcomes of pregnant women with dengue infection were stillbirth (crude relative risk: 6.7), miscarriage (odds ratio: 3.51), preterm birth (odds ratio: 1.71), and low birth weight (odds ratio: 1.71) [12]. A miscarriage was recently reported to occur in a pregnant woman infected by dengue virus, and DENV RNA was detected in fetal material [13]. Thus, dengue infection in pregnant women should undoubtedly be taken seriously.

## 3. Initial Immune Response to Dengue in Pregnant Women

Dengue fever is widely accepted as an immunopathological disease. Thus, the pathogenesis of dengue can be explained by various mechanisms related to immune factors as follows: (1) enhancement of viral infection through cross-reactive antibodies; (2) activation of cross-reactive memory T cells; (3) a cytokine or inflammatory storm; and (4) complement activation. However, a very large part of dengue pathogenesis remains elusive despite these arduous studies. Since dengue is an immune-related disease, whether infection becomes limited or progressive depends on the competition between defensive and destructive immune responses.

The initial immune response to viral infection is mediated by interferons (IFNs) [14]. IFNs have displayed increasing diversity and activity to “interfere” with viral replication. IFNs are divided into three types: type I mainly represented by IFN-*α* [15] and IFN-*β* [16], type II represented by INF-*γ* [17], and type III including the recently discovered IFN-lambda (IFN-*λ*) family [18]. Each IFN family member mediates important antiviral activity via engagement with its specific IFN receptor.

### 3.1. IFN Level in Dengue Infection

Few studies have evaluated serum IFNs level in pregnant women during dengue infection. However, a larger number of studies have explored serum IFN levels in dengue patients. For type I IFNs, the serum level of IFN-*β* was significantly higher in primary DHF patients than in patients with dengue fever [19], suggesting that high levels of IFN-*β* might accompany a worsened progression of the disease. However, the other type 1 IFN, IFN-*α*, displayed an opposite phenomenon. The serum IFN-*α* level was significantly higher in DF patients than in DHF patients, irrespective of the DENV serotypes [20], and the dynamic change of serum level significantly reduced in 3–5 days after fever onset (DENV-1: reduced from 94.42 pg/mL to 36.12 pg/mL; DENV-2: reduced from 53.39 pg/mL to 38.25 pg/mL). Also the serum level of IFN-*α* was significantly higher in dengue patients than in healthy individuals [21]. These findings suggest that the early robust production of IFN-*α* may be correlated with a better clinical condition with respect to dengue infection and disease progression. Thus, the roles of type I IFNs in dengue infection are contradictory and complicated.

For type II IFNs, the serum level of IFN-*γ* was higher in dengue patients than in healthy individuals [22]. The same finding was also reported by Feitosa et al. [23]. The serum level of IFN-*γ* was gradually decreased in 3–5 days, 6-7 days, 8–10 days, and 14–17 days since illness onset in both mild and severe dengue patients [24], and it was higher in severe dengue patients in 8–10 days after illness onset compared with the mild dengue patients, but no difference was found in the other times between these two groups [24]. This observation was confirmed by a cohort study reported by Cui et al. [25]. However, the higher level of IFN-*γ* was found in patients within 96 hours from fever onset. According to these findings, the high concentration of IFN-*γ* in dengue patient serum may indicate a high risk of disease progression to severe dengue.

For type III IFNs, there are few clinical studies reporting mild or severe dengue. Nevertheless, in vitro studies found that dengue virus infection could induce the production of IFN-*λ* [26]. An elevated serum IFN-*λ* level was also observed in patients with dengue fever, compared to healthy blood donors, and IFN-*λ* could inhibit dengue virus replication in vitro [27].

In general, a high serum level of IFNs in dengue patients accompanies a high risk of disease progression to severe dengue. This raises the question of whether lower serum IFN level indicates a lower risk of disease progression to severe dengue. The answer to this question is “no.” It has been reported that the mean level of serum IFN-*γ* in DF cases was higher than that in DHF patients, which implied that low serum IFN-*γ* level might be associated with severe diseases [28]. A Recent research has shown that the serum IFN-*γ* concentration in dengue patients is negatively correlated with the dengue virus load, indicating that a lower the serum level of IFN-*γ* is correlated with a higher dengue viral load in dengue patients [29]. Although dengue virus is regarded as an immunopathological disease, it is also dire [30]. Additionally, a high viral load is one important cause of severe dengue because the virus can destroy host cells and lead to vascular leakage, which accelerates disease progression in a short period of time [31, 32]. Thus, one strategy to reduce the possibility of disease progression to severe dengue is to decrease the dengue viral load. Therefore, a high level of type II IFNs is needed for the treatment of dengue patients in clinics. It has been also reported that, during acute infection, higher serum levels of IFN-*β* [6.69 (0.15~34.9) pg/mL] were found in DHF patients with primary infection than that [2.35 (0~10.1) pg/mL] in the DHF patients with secondary infection [19]. To this point, the serum IFN-*β* level of dengue patients was not associated with dengue progression.

### 3.2. IFN Level in Pregnant Women

IFNs are expressed by the trophectoderm of primates, rodents, and ungulates during peri-implantation [33]. Conceptus IFNs are pregnancy signals for maternal recognition in ruminants (cattle, sheep, and goats); they act on the endometrium to indirectly maintain progesterone synthesis [34]. Additionally, studies investigating IFN signaling in the uterus of livestock species suggest that it modulates maternal immune tolerance to the implanting conceptus, changes in the endometrial architecture for uterine receptivity, and vascular remodeling for maternal-fetal nutrient and waste exchange [35, 36]. Although these findings for IFNs have rarely been reported in humans, they are still useful references for pregnant women. Although few studies have described the outcomes of pregnancy with dengue and the relationship between IFNs and pregnancy after DENV infection, such findings have been extensively reported for Zika virus, which belongs to the same family of Flaviviruses as DENV. In a mouse model, type I IFNs could control vaginal Zika virus replication [37]. In vitro, Zika virus infection has been shown to cause the constitutive release of IFN-*λ*, the antiviral type III IFN, by human placental trophoblast cells [38]. These findings raise the question of whether a higher level of IFNs is beneficial for pregnant women with dengue infection. The answer to this question is also “no.” In an in vitro embryo culture study, IFN-*β* showed no macroscopic teratogenic effect on embryonic development, but it caused growth retardation in embryos [39].

Li et al. also showed that IFN-*γ* could induce pregnancy failure by moderating natural killer (NK) cells [40], and it could dramatically increase decidual apoptosis [41]. In a case control study, women with extremely low birth weight infants with accepted induced spontaneous conception or assisted reproductive technology showed increased production of IFN-*γ* [42]. Furthermore, IFN use in pregnant women is not advised, especially given its known antiproliferative effects [43]. A cohort study has indicated that IFN therapy during the first trimester of pregnancy can lead to a high risk of fetal loss and low birth weight [44]. Animal trials have demonstrated that IFN-*γ* administration can result in pregnancy failure [45, 46]. Thus, in pregnant women, IFNs should be maintained at a certain level for a stable pregnancy.

### 3.3. The Mechanism by Which IFNs Induce Adverse Pregnancy Outcomes

Most adverse pregnancy outcomes are associated with pathogen infection, although the precise mechanism leading to adverse outcomes via infection is unknown. One established factor is the specific immune response to pathogen infection in pregnant women. Whether IFNs, particularly IFN-*γ*, play a positive or negative role during pregnancy has raised numerous controversies. For other IFN family members, few studies have focused on their influence on pregnant women with dengue virus. Our knowledge is derived mostly from animal or in vitro experimental studies, because data for human pregnancies are scarce.

In vitro, IFN-*γ* is cytotoxic to human trophoblast cells [47] and inhibits their proliferation [48]. An in vitro experiment showed that the levels of IFN-*γ* secreted by decidual NK cells were closely correlated with trophoblasts apoptosis in response to* Toxoplasma gondii* infection [49]. Comba et al. reported that the levels of IFN-*γ* in blood and endometrial tissue were significantly higher in patients with recurrent pregnancy loss than in women with normal fertility [50].

IFN-*γ* can also lead to adverse pregnancy outcomes through the following signaling pathways. In a mouse model to induce fetal resorption through the injection of *α*-galactosylceramide (*α*GC), decidual invariant NK T cells (iNKT) were activated followed by the upregulation of IFN-*γ* levels. This adoptive change resulted in pregnancy loss in IFN-*γ*^−/−^ mice [51]. One signal transduction pathway leads to fetal mortality is class I phosphoinositide 3-kinase (PI3K), which converts phosphatidylinositol-(4,5)-phosphate to phosphatidylinositol-(3,4,5)-phosphate (PIP3). Acting as a second messenger, PIP3 recruits proteins to the plasma membrane, where they activate signaling pathways that promote cell proliferation, survival, and differentiation [52]. Based on structural similarity, PI3K can be divided into two classes: class IA and class IB. Class IA PI3K forms heterodimers of p85 regulatory subunits and one of the three isoforms of the catalytic p110 subunit (p110-*α*, p110-*β*, and p110-*δ*). PI3K p110-*δ* is a key mediator of NK cell maturation and function. An absence of p110-*δ* signaling leads to reduced cytokine release, aberrant maturation, and incorrect trafficking to peripheral organs, including the uterus during pregnancy [53, 54]. When PI3K and p110-*δ* were deactivated, the uterus showed a decreased level of IFN-*γ* and elevated IL-6, resulting in fetal death or growth retardation [55]. Simultaneously, IFN-*γ* can exert feedback control on Ly-49 receptors to regulate NK cell effector functions during pregnancy failure [40]. The other pathway is the Notch signaling pathway, which exerts its effects throughout pregnancy, playing an important role in placental angiogenesis and trophoblast function [56]. During peptidoglycan and polyinosinic:cytidylic acid-induced preterm labor, Notch signaling is activated, resulting in the upregulation of proinflammatory responses with upregulated levels of IFN-*γ*, TNF-*α*, and IL-6, and its inhibition improves the in utero survival of live fetuses [57]. In general, the mechanism by which IFNs induce adverse pregnancy outcomes is complicated and requires further study.

## 4. Conclusion

Dengue is a serious public health problem and also a threat to the pregnant women who have a higher risk of progressing to severe dengue or experiencing adverse pregnancy outcomes after infection. The initial immune response to dengue virus infection, especially with respect to IFN production, was reviewed in this group of patients. Dengue is much more prevalent in pregnant women in comparison to the other groups. The severity of dengue is correlated with the high level of some IFNs in patient' serum, while the serum IFN level must be maintained at a sufficiently high level to maintain the pregnancy and inhibit dengue virus replication. However, many of the above-described findings represent only clinical phenomena or were derived from in vivo/in vitro animal experiments. The effects of IFNs on human pregnancies are more difficult to study. Hence, further analyses are needed to reach an unambiguous conclusion regarding the roles of IFNs during dengue virus infection, especially in pregnant women.

## Abbreviations

DENV: Dengue virus
DHF: Dengue hemorrhagic fever
DSS: Dengue shock syndrome
DF: Dengue fever
IFNs: Interferons
NK: Nature killer
*α*GC: *α*-Galactosylceramide
iNKT: Invariant natural killer T cells
PI3K: Phosphoinositide 3-kinases
PIP3: Phosphatidylinositol-(4,5)-phosphate to phosphatidylinositol-(3,4,5)-phosphate.
